# Oral fibroblasts modulate the macrophage response to bacterial challenge

**DOI:** 10.1038/s41598-017-11771-3

**Published:** 2017-09-14

**Authors:** Rinat Tzach-Nahman, Rizan Nashef, Omer Fleissig, Aharon Palmon, Lior Shapira, Asaf Wilensky, Gabriel Nussbaum

**Affiliations:** 1 The Institute of Dental Sciences, the Hebrew University-Hadassah Faculty of Dental Medicine, Jerusalem, Israel; 2 The Department of Periodontology, the Hebrew University-Hadassah Faculty of Dental Medicine, Jerusalem, Israel; 3 The Department of Oral and Maxillofacial Surgery, the Hebrew University-Hadassah Faculty of Dental Medicine, Jerusalem, Israel; 4 The Department of Orthodontics, the Hebrew University-Hadassah Faculty of Dental Medicine, Jerusalem, Israel

## Abstract

Tissue damage in chronic periodontal disease is driven by the host response to a dysbiotic microbiota, and not by bacteria directly. Among chronic inflammatory diseases of the oral cavity, inflammation and tissue damage around dental implants (peri-implantitis) is emerging as a major clinical challenge, since it is more severe and less responsive to treatment compared to inflammation around natural teeth. We tested whether oral fibroblasts from the periodontal ligament (PDLF), which are present around natural teeth but not around dental implants, actively regulate inflammatory responses to bacterial stimulation. We show that human PDLF down-regulate TNF-α post-transcriptionally in macrophages stimulated with the oral pathogen *Porphyromonas gingivalis*. Cell contact and secretion of IL-6 and IL-10 contribute to the modulation of inflammatory cytokine production. Although fibroblasts decreased TNF-α secretion, they enhanced the ability of macrophages to phagocytose bacteria. Surprisingly, donor matched oral fibroblasts from gingival tissues, or fibroblasts from peri-implant inflamed tissues were at least as active as PDLF in regulating macrophage responses to bacteria. In addition, priming fibroblasts with inflammatory mediators enhanced PDLF regulatory activity. A further understanding of the spectrum of fibroblast activities in inflammatory lesions is important in order to design ways to control inflammatory tissue damage.

## Introduction

Periodontal disease involves a combination of bacterial infection and an ineffective host immune response which leads to periodontal tissue destruction and potential tooth loss^1^.

A substantial part of the tissue damage that characterizes periodontal disease can be attributed to the host response to infection, rather than direct pathogen effects^2^. For example, localized aggressive periodontitis correlates to excessive neutrophil activity and release of toxic products that drive tissue destruction^3^. In chronic periodontitis, the resolution phase that normally follows acute inflammation becomes disordered, leading to the persistence of the inflammatory infiltrate and to tissue destruction. Such a disturbance in the resolution of the acute phase of inflammation may be related to the behavior of stromal cells, which play a key role in the transition from innate to adaptive immunity^4^. By actively conditioning the local cellular and cytokine environment, stromal cells may lead either to the maintenance of homeostasis^5^ or to progression to chronic inflammation^6^. In the case of the periodontal stroma, the major cellular component is the fibroblast, comprising 65 percent of the cells in the gingival connective tissue^7^. By supplying signals such as cytokines and chemokines, fibroblasts may guide immune cell behavior, and therefore their phenotype can determine the outcome of the inflammatory infiltrate during periodontal disease^4^.

One of the possible outcomes of periodontal disease is the loss of teeth due to a critical decrease in periodontal tissue support. The use of dental implants to compensate for tooth loss is increasing annually^8^, however bacterial infection and tissue inflammation is an emerging problem that can lead to peri-implant mucositis and eventually to peri-implantitis which includes resorption of the peri-implant bone leading to loss of the implant^9^. Peri-implantitis is more extensive, destructive, and progresses faster, than chronic periodontitis^10^, and peri-implantitis is also less responsive to treatment^9^.

One major difference between natural teeth and implants is in the integration to alveolar bone of the jaw. Natural teeth are anchored to alveolar bone through the periodontal ligament (PDL), a 0.15–0.38 mm connective tissue composed mainly of ordered fibroblasts and their extracellular fibrous matrix^11^. In contrast, implants directly integrate within the osseous matrix of alveolar bone^12^. Traumatic tooth injuries can also lead to loss of the periodontal ligament, resulting in ankylosis of natural teeth, and direct integration to alveolar bone in a similar fashion to what occurs with implants. Interestingly, the inflammatory process around ankylosed teeth is more severe, and resembles peri-implantitis in the extent of tissue damage^13^. Thus, the PDL appears to limit the extent and severity of mucosal inflammation. We hypothesized that fibroblasts originating from the PDL (periodontal ligament fibroblasts, PDLF) play an active immunomodulatory role, leading to the more limited inflammatory process around natural teeth in comparison to dental implants. We aimed to test whether PDLF modulate inflammation more than gingival fibroblasts (GF) which are present around dental implants. Our goal was to understand the ability of oral fibroblasts to influence the innate immune response towards periodontal pathogens, and to examine whether PDLF are more modulatory than GF, which may explain the more destructive nature of peri-implantitis in comparison to periodontitis.

## Results

### PDLF down-regulate macrophage cytokine production in response to *P*. *gingivalis*

**Figure 1:**
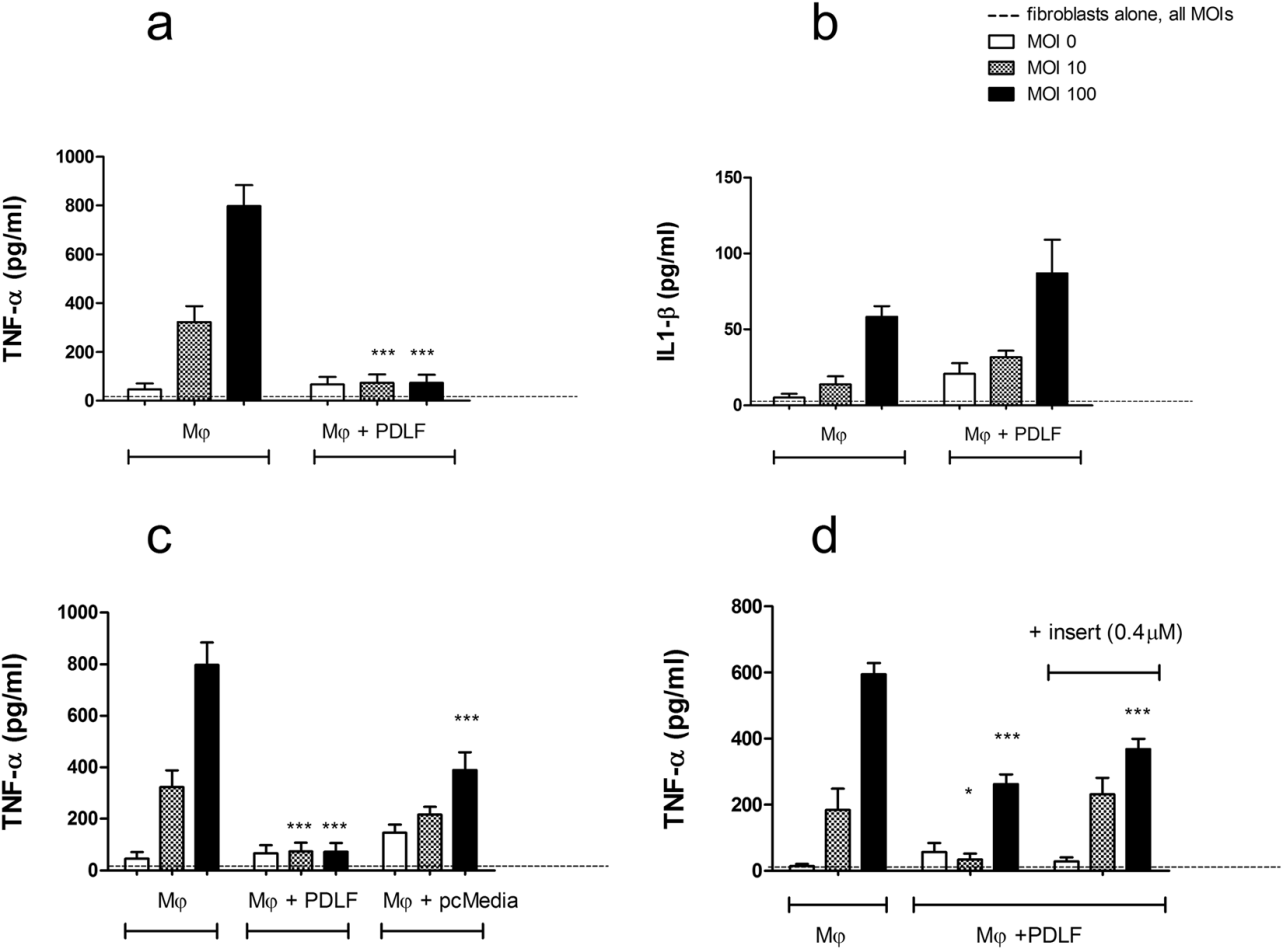
PDLF down-regulate macrophage cytokine production in response to *P*. *gingivalis*. Human macrophages (Mφ) were mono or co-cultured with PDLF prior to stimulation with increasing MOIs of *P*. *gingivalis*. TNF-α (**a**) and IL1-β (**b**) production were measured in the supernatant 5 hours after bacterial stimulation. Contact dependency of the regulatory effect of PDLF over the TNF-α production by stimulated Mφ was tested using PDLF pc-media (**c**) or a 0.4 μm transwell chamber (**d**) separating the two cell populations. Data represent the means + SEM of at least 3 separate experiments. *P value < 0.05 compared to the cytokine production by Mφ in mono-culture stimulated with the same MOI. ***P < 0.001 compared to the cytokine production by Mφ in mono-culture stimulated with the same MOI.

To examine the influence of PDLF on the inflammatory response of macrophages to oral bacteria, human macrophages were cultured alone vs. together with increasing ratios of PDLF or their pre-conditioned (pc) media before stimulation with *P*. *gingivalis*. Following bacterial stimulation (5 h), release of pro- and anti-inflammatory cytokines was measured. Macrophages secreted TNF-α, a pro-inflammatory cytokine important in the pathogenesis of the tissue destruction during periodontal disease^14^, in a dose dependent manner in response to stimulation with *P*. *gingivalis* (Fig. 1a). In the presence of PDLF, TNF-α secretion was significantly decreased (P value < 0.001) and remained minimal even at increasing multiplicity of infection (MOI) of the bacteria (Fig. 1a). Macrophage TNF-α secretion was significantly lowered even at the lowest ratio of PDLF tested (Supplementary Fig. S1). Fibroblasts stimulated with bacteria, did not secrete detectable levels of TNF-α. Macrophage secretion of IL-1β, a cytokine related to periodontal disease exacerbation and alveolar bone loss^15^, increased in response to bacterial stimulation and reached significance in MOI 100 (P value < 0.01). In contrast to TNF-α, co-culture of macrophages with PDLF did not decrease IL-1β secretion (Fig. 1b).

We next asked whether contact between the fibroblasts and macrophages is required in order to down-regulate the macrophage TNF-α response to bacteria. We found that the pc-media of PDLF significantly lowered TNF-α secretion in response to MOI 100 of the bacteria (P value < 0.001) (Fig. 1c). Compared to co-culture with the PDLF cells, the effect of their conditioned medium was partial and only observed in response to the highest MOI. Therefore, PDLF secrete factors constitutively that modulate macrophage responses, however physical contact with the macrophages enhances the regulatory effect. To confirm that contact is truly required for the maximal effect, we used 0.4 µM transwell chambers to separate the two cell populations. In this setting, the modulatory effect of PDLF co-culture was only partial (Fig. 1d), demonstrating that both contact-dependent and contact-independent processes contribute to the modulatory effect of PDLF on macrophages responding to *P*. *gingivalis*.

### TNF-α regulation by PDLF is post-transcriptional

**Figure 2:**
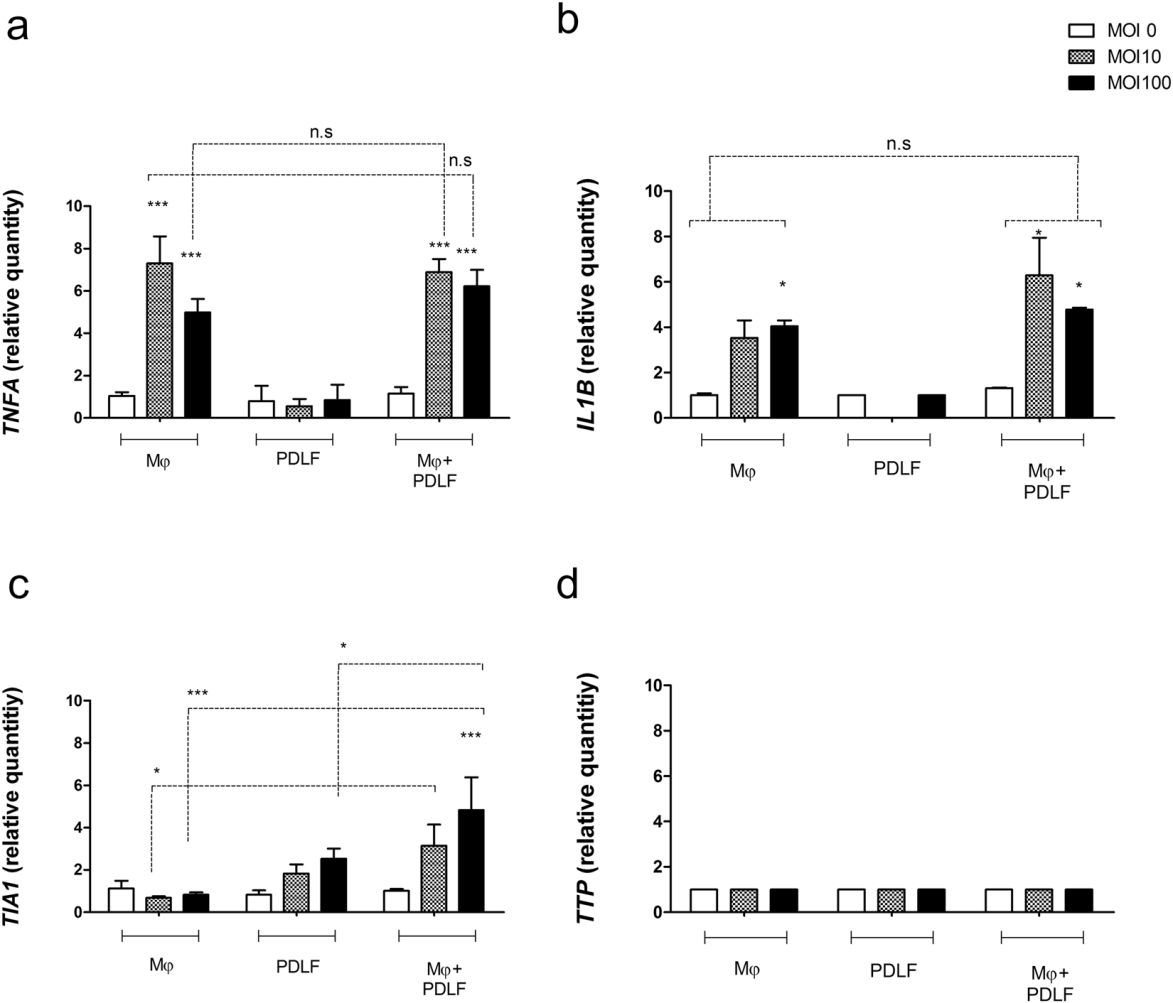
TNF-α regulation by PDLF is post-transcriptional. Human macrophages (Mφ) were mono or co-cultured with PDLF prior to stimulation with increasing MOIs of *P*. *gingivalis*. *TNFA* (**a**), *IL1B* (**b**), *TIA-1* (**c**) and *TTP* (**d**) levels were measured by qRT-PCR 5 hours after bacterial stimulation and normalized to the level of *HBA*. Relative quantity of each transcript at the indicated MOI was compared to MOI 0 in the same culture type. Data represent means + SEM of at least 3 separate experiments. Transcript levels were compared to the level in MOI 0 of the same culture, and between culture types at the same MOI. *P < 0.05; ***P < 0.001; n.s = not significant.

To investigate the differential regulation of TNF-α and IL1-β, we tested the effect of co-culturing human macrophages with fibroblasts on the mRNA of *TNFA* (Fig. 2a) and *IL1B* in response to *P*. *gingivalis* stimulation (Fig. 2b). As expected, *IL1B* mRNA was induced by *P*. *gingivalis*, and the level was not affected by co-culture with fibroblasts. Surprisingly, in contrast to the reduction in TNF-α protein production, up-regulation of *TNFA* mRNA was not affected by the presence of the fibroblasts. Thus, the regulation of TNF-α by fibroblasts is post-transcriptional. To elucidate the potential mechanism of post-transcriptional regulation of TNF-α, we focused on mRNA binding proteins that differentially regulate the *TNFA* ^16–18^ transcript vs. factors that regulate both *TNFA* and *IL1B* ^19^. Intriguingly, co-culture with fibroblasts led to an increase in the level of T-Cell-Restricted Intracellular Antigen-1 (*TIA1*) (Fig. 2c), an mRNA binding protein that specifically binds to AU-rich elements located in the 3′ untranslated region of the *TNFA* transcript and suppresses its translation. In contrast, co-culture did not up-regulate the levels of Tristetraprolin (*TTP*) (Fig. 2d), an RNA binding protein shown to repress the transcripts of both *TNFA* and *IL1B*.

### IL-6 and IL-10 contribute to the modulation of TNF-α secretion by macrophages responding to bacterial stimulation

**Figure 3:**
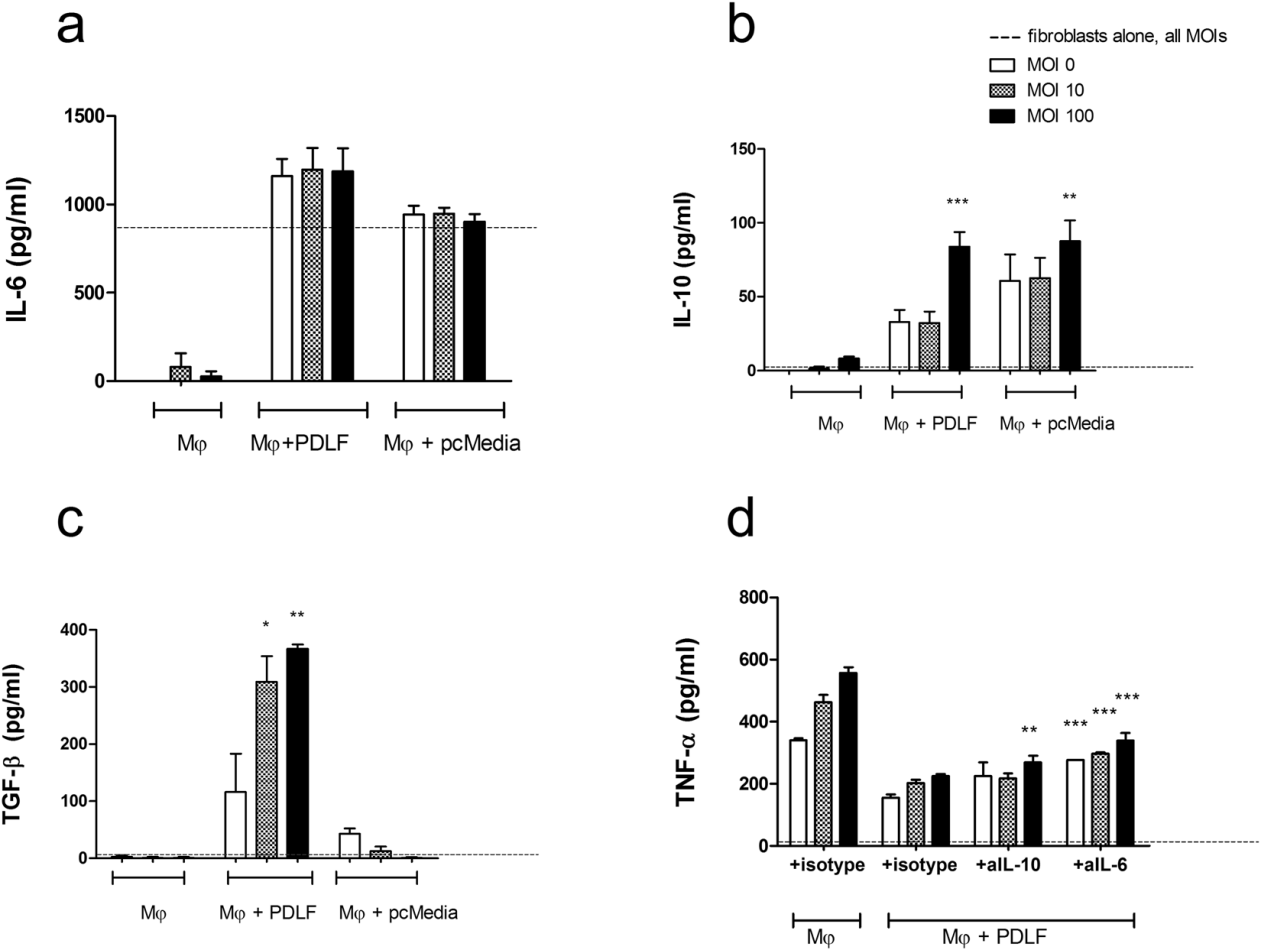
IL-6 and IL-10 contribute to the modulation of TNF-α secretion by stimulated Mφ. Human macrophages (Mφ) were mono or co-cultured with PDLF prior to stimulation with increasing MOIs of *P*. *gingivalis*. IL-6 (**a**), IL-10 (**b**) and TGF-β (**c**) production were measured in the supernatant 5 hours after bacterial stimulation. Dashed line represents cytokine secretion by mono-cultured fibroblasts. Blocking of IL-10 or IL-6 in co-cultures of Mφ and PDLF decreased the modulatory effect over TNF-α production following stimulation with *P*. *gingivalis* (**d**). Data represent the means + SEM of at least 3 separate experiments. **P value < 0.01 compared to the cytokine production by Mφ in mono-culture stimulated with the same MOI (**b**) or to co-cultures of Mφ and PDLF treated with isotype control and the same MOI (**d**). *P < 0.05; **P < 0.01; ***P < 0.001 compared to the cytokine production by macrophages in mono-culture stimulated with the same MOI (a,b,c), or to co-cultures of Mφ and PDLF treated with isotype control and the same MOI (**d**).

To better understand the factors that lead to modulation of the macrophage response, we next examined the production of IL-6, IL-10, and TGF-β by each cell type alone, or in the co-culture system. We found spontaneous IL-6 production in the PDLF media, and increased production when macrophages were added to the PDLF even without bacterial stimulation (Fig. 3a). IL-6 secretion was not detected in mono-cultures of macrophages and was not affected by their stimulation with *P*. *gingivalis* under these conditions. In contrast to the spontaneous secretion of IL-6 by PDLF, IL-10, an anti-inflammatory cytokine that is able to inhibit TNF-α secretion in LPS stimulated monocytes and macrophages^20, 21^, is not produced by PDLF or macrophage mono-cultures (either unstimulated or stimulated with *P*. *gingivalis*, Fig. 3b). Co-culture of macrophages with PDLF or exposure to PDLF pc-media, induces IL-10 secretion in small amounts that increase with exposure to increasing *P*. *gingivalis* MOIs (Fig. 3b). In contrast to both IL-6 and IL-10, TGFβ production required the combination of macrophages, PDLF, and bacteria (Fig. 3c).

To examine if the fibroblast modulatory activity depended on IL-6 and IL-10, we next blocked both cytokines using inhibitory antibodies independently or simultaneously. We chose not to block TGFβ since fibroblasts without modulatory activity in this system (discussed below) also displayed high TGFβ, but not IL-6 and IL-10. Inhibition of IL-6 or IL-10 alone, partially reduced the fibroblast modulatory effect, with the effect of blocking IL-6 being more substantial than that of IL-10 (Fig. 3d). Simultaneous inhibition of both cytokines did not further reduce the modulatory activity over the effect of blocking IL-6 alone (Fig. 3d).

### PDLF increase macrophage phagocytosis of *P*. *gingivalis*

**Figure 4:**
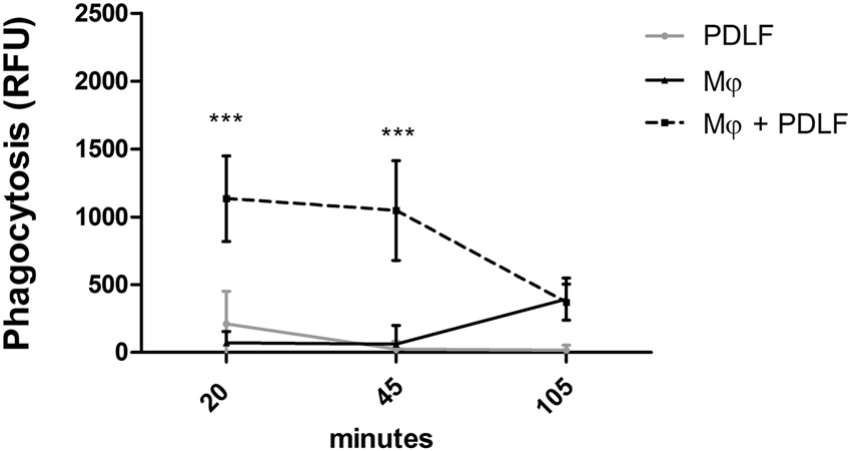
PDLF increase Mφ phagocytosis of *P*. *gingivalis*. Mono-cultures or co-cultures of Mφ and PDLF were infected with FITC-labeled *P*. *gingivalis* for up to 105 minutes. Following incubation, cells were thoroughly washed and quenched with trypan blue. Phagocytosis was assessed using a fluorescence reader, and data are shown as relative fluorescence units (RFU). Data represent the means + SEM of at least 5 separate experiments. ***P < 0.001 between Mφ in mono-culture and Mφ in co-culture with PDLF at the tested time point.

Decreased macrophage inflammatory cytokine production may adversely impact on the phagocytic response^22, 23^. To address this question, we challenged human macrophages with fluorescently-labeled *P*. *gingivalis* in the presence or absence of PDLF, and followed phagocytosis (Fig. 4). PDLF alone did not internalize fluorescently-labeled bacteria under these conditions. In contrast, macrophages cultured alone phagocytosed *P*. *gingivalis*, and their phagocytosis was significantly enhanced when they were co-cultured with PDLF, even at the earliest time point tested (20 minutes post challenge). Maximal phagocytosis by macrophages cultured in the absence of PDLF was significantly less than in the presence of PDLF. Therefore, the decreased cytokine production induced by PDLF correlates with enhanced, rather than decreased, macrophage phagocytosis following bacterial challenge.

### Fibroblasts down regulate SDF-1α mediated monocyte migration

**Figure 5:**
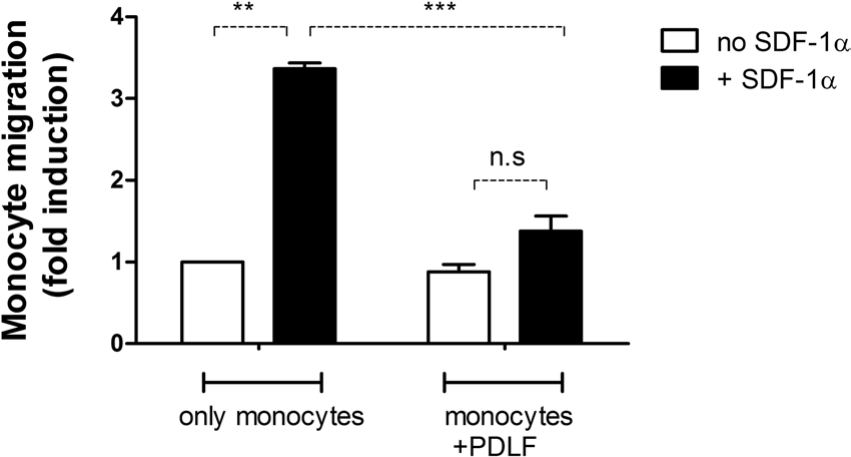
Fibroblasts down regulate SDF-1α mediated monocyte migration. CFSE labeled human monocytes were migrating in a 5 mm transwell chamber towards a gradient of SDF-1α, either with or without PDLF in the bottom chamber. After 3 hours of migration, CFSE positive cells in the upper and the lower chambers were counted by FACS. Fold induction of migration was calculated in comparison to spontaneous migration of monocytes in mono-culture without the presence of an SDF-1α gradient in the chamber. Data represent means + SEM of 3 different experiments. **P < 0.01; ***P < 0.001; n.s = not significant.

In peri-implantitis, the inflammatory cell infiltrate is larger than in periodontitis, and it extends deeper within the soft tissue, reaching the bone^24^. To determine the contribution of PDLF to this difference, we tested whether PDLF decrease monocyte migration, using a transwell chamber system (Fig. 5). Since bacterial challenge did not induce monocyte migration, we tested migration in response to an SDF-1α gradient. The chemokine SDF-1α is expressed by endothelial cells in response to tissue damage and increases during periodontal disease, both clinically and in experimental periodontitis following challenge with *P*. *gingivalis* ^25^. As expected, SDF-1α strongly induced human monocyte migration over a period of 3 hours relative to spontaneous migration. However, when PDLF were present in a monolayer on the surface of the bottom chamber, monocyte migration in response to SDF-1α was completely abrogated. To rule out a potential confounding effect due to PDLF inactivation of SDF-1α (either by absorption or other means), we cultured SDF-1α for 3 hours with PDLF monolayers (versus no PDLF), and then compared the effect on monocyte migration. Pre-incubation of SDF-1α with PDLF did not affect its activity. Therefore, in addition to affecting the activation state of the macrophage, PDLF are able to reduce monocyte migration in response to a chemokine gradient.

### HGF cell line, but not donor matched primary GFs, is less modulatory than PDLF

**Figure 6:**
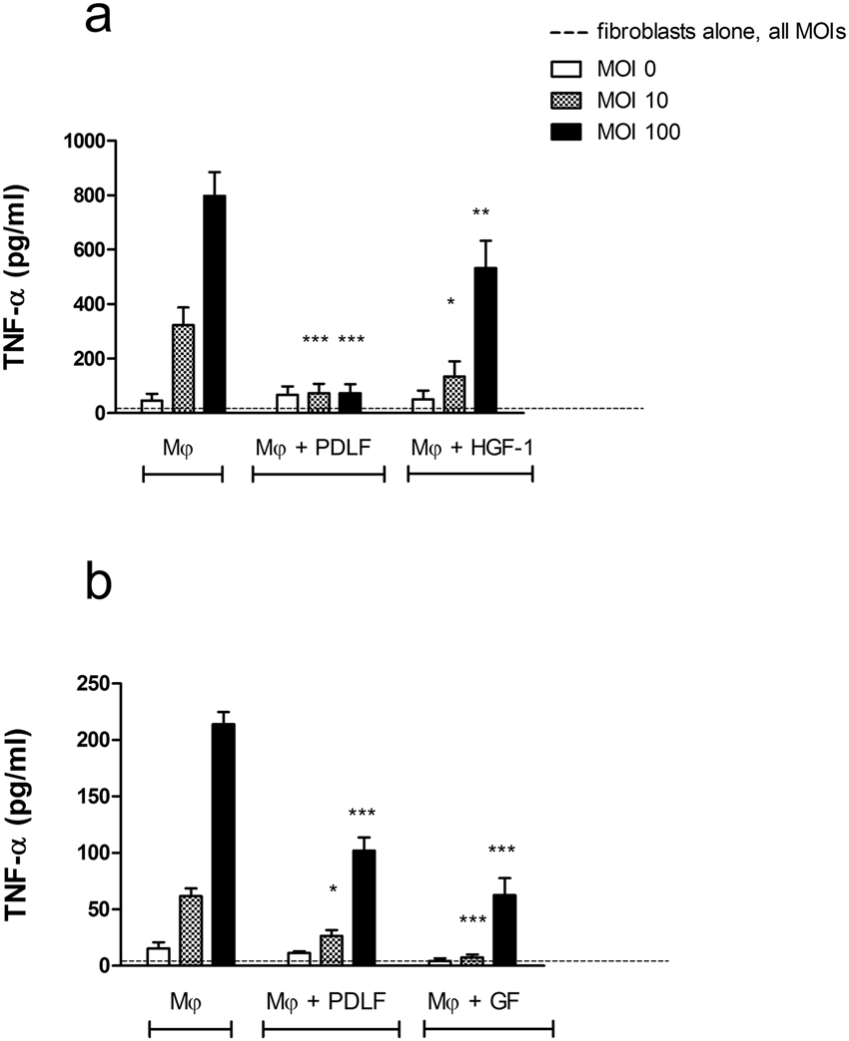
HGF cell line, but not donor matched primary GFs, is less modulatory than PDLF. Human Mφ were cultured alone or together with PDLF, or an HGF-1 cell line (**a**). Separately, Mφ were cultured alone or together with primary GF vs. PDLF from the same or different donors (**b**). Cells were stimulated with increasing MOIs of *P*. *gingivalis* and TNF-α production was tested in the supernatant after 5 hours. Data represent the means + SEM of at least 3 separate experiments. *P < 0.05; **P < 0.01 and ***P < 0.001 in comparison to the cytokine production by Mφ in mono-culture stimulated with the same MOI.

To test if the effect of PDLF on monocyte/macrophages is specific to fibroblasts originating from the periodontal ligament, we first compared the effect to that of an immortalized human GF cell line (HGF-1, ATCC). HGF-1 cells were significantly less effective than PDLF in reducing TNF-α secretion in bacterially stimulated co-cultures of human macrophages and fibroblasts (P value < 0.01, Fig. 6a). In addition, the pc-media of resting HGF-1 did not affect TNF-α secretion by stimulated macrophages, in contrast to the pc-media of PDLF (Supplementary Fig. S3).

To compare primary GF to the primary PDLF, we next established a pool of GF and PDLF, some of which were derived from the same donors. Healthy donors scheduled for extraction of wisdom teeth or extraction of premolars prior to orthodontic treatment were consented. PDLF were derived from the middle third of the root of the tooth extracted. Gingival fibroblasts were derived from the same donors, by collecting 1 mm^3^ fragments of free gingiva that were surrounding the extracted teeth and removed in order to shape the soft tissue to enhance its healing. Consistent with published reports, PDLF from all donors expressed higher levels of TGFβRII than the GF populations (Supplementary Fig. S4), validating that we established distinct fibroblast populations. In contrast to our findings with the HGF-1 line, primary GF demonstrated similar to greater modulatory effects on activated macrophages than PDLF (either donor-matched or not donor matched, Fig. 6b). This finding was consistent over all MOIs tested, and between all the donors, and led us to speculate that the environment of the GF primed them to be more modulatory.

### Inflammation increases the modulatory activity of oral fibroblasts

**Figure 7:**
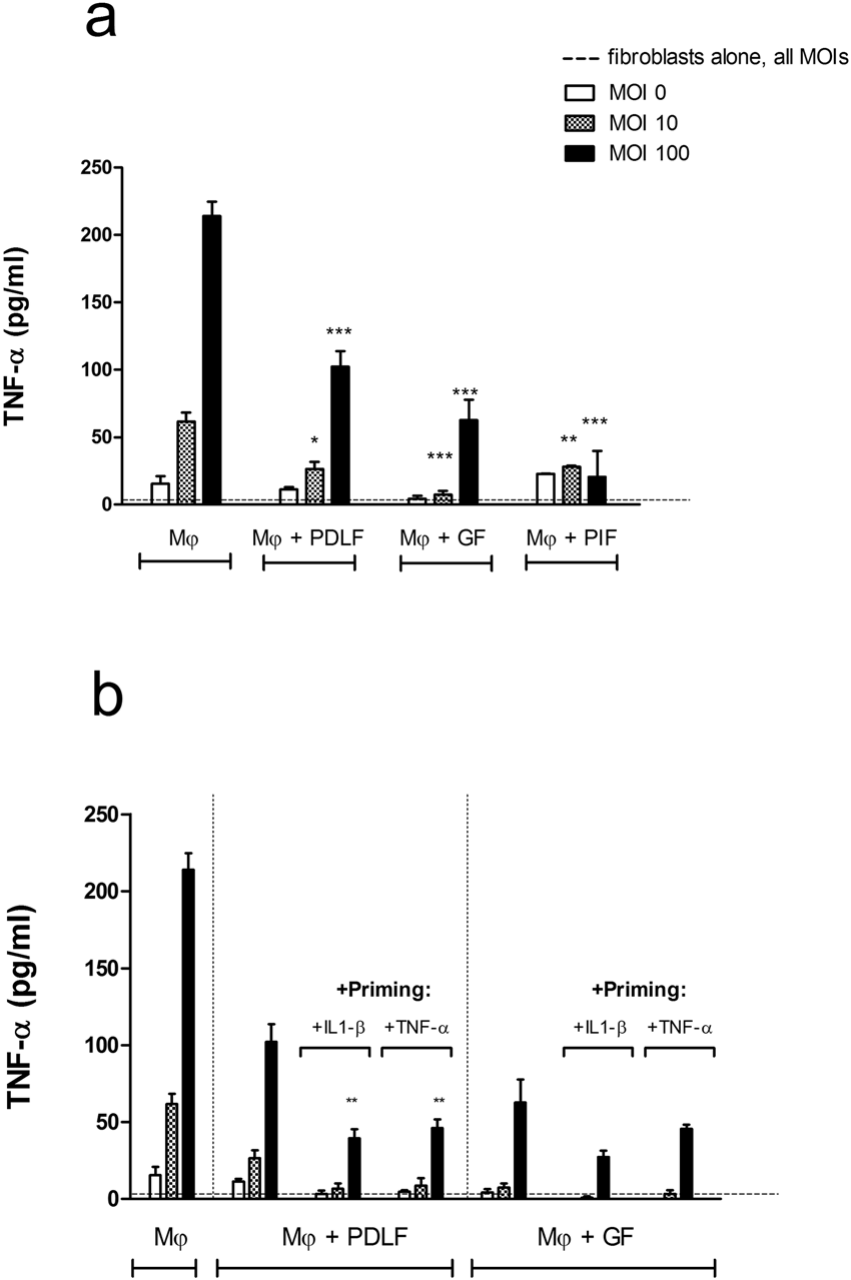
Inflammation increases the modulatory activity of oral fibroblasts. Mφ were cultured alone or together with primary PDLF, GF and PIF (**a**) or with primary PDLF or GF primed with IL1-β or TNF-α for 3 days. TNF-α production was tested following stimulation with *P*. *gingivalis* for 5 hours. Data represent the means + SEM of at least 3 separate experiments. *P < 0.05; **P < 0.01 and ***P < 0.001 in comparison to the cytokine production by Mφ in mono-culture stimulated with the same MOI (**a**), or to cytokine production by Mφ co-cultured with un-primed fibroblasts and stimulated with the same MOI.

In contrast to PDLF, GF are derived from a tissue in constant contact with microbes, and even a clinically normal un-inflamed gingival tissue harbors a leukocytic infiltrate that responds to the adjacent bacterial biofilm^26^. We hypothesized that the modulatory activity of fibroblasts is enhanced by inflammation itself. We therefore next tested the modulatory activity of primary fibroblasts derived from overtly inflamed peri-implant mucosal tissue (PIF). To harvest PIF, subjects suffering from peri-implantitis who were scheduled for scaling or implant removal were recruited to the study. Fibroblasts derived from peri-implantitis granulation tissue demonstrated the strongest modulatory activity of all the human oral fibroblasts tested (Fig. 7a). This was true for PIF at both early and late passage numbers (Supplementary Fig. S5). We next asked whether priming of fibroblasts with inflammatory cytokines associated with periodontal disease would increase their modulatory activity. In the case of PDLF, cells that were primed with either IL1-β or TNF-α (10 ng/ml) for 3 days (followed by extensive washing) demonstrated maximal regulation of TNF-α secretion levels relative to co-cultures of macrophages with untreated PDLF (Fig. 7b).

## Discussion

Our principal finding is that primary human oral fibroblasts decrease the inflammatory cytokine production of macrophages exposed to *P*. *gingivalis*. TNF-α, an important cytokine related to bone loss in chronic periodontal disease^14, 27^, was strongly downregulated in the presence of fibroblasts. The modulatory activity of the primary oral fibroblasts was enhanced in an infected and inflamed environment (peri-implantitis). Since fibroblasts modulate the innate inflammatory response of macrophages non-specifically (Supplementary Fig. S4), we propose to call this activity “inflammomodulatory,” to distinguish it from immunomodulatory activities that occur in the context of antigen-specific responses. We demonstrated that TNF-α secretion was downregulated in the presence of PDLF, however IL1-β production in response to bacterial infection was not significantly reduced. The differential regulation of TNF-α secretion by fibroblasts is post-transcriptional, since *TNFA* mRNA transcript levels were induced in macrophages to a similar degree in the absence or presence of fibroblasts. Of interest, co-culture with fibroblasts led to an increase in the levels of the mRNA binding protein *TIA1*, which specifically regulates the *TNFA* transcript^16, 17^, while the levels of *TTP*, which repress the transcripts of both *TNFA* and *IL1B* ^19^ were not upregulated by co-culture with fibroblasts. Overall, the differential regulation of TNF-α vs IL1-β is consistent with other reports that show these cytokines can be differentially regulated^28, 29^, however, the mechanisms governing regulation by fibroblasts require further investigation.

After demonstrating the inflammomodulatory activity of PDLF, an important question became whether fibroblasts negatively impact the macrophage phagocytic response to bacterial challenge. Surprisingly, we saw that the interaction of PDLF and human macrophages lead to improved macrophage phagocytosis of live *P*. *gingivalis*. Improved bacterial phagocytosis by macrophages in the presence of PDLF was also observed by Konermann *et al*.^30^, and the findings are consistent with the report of Smitheys *et al*., who showed that intestinal stromal cell products strongly reduce macrophage inflammatory cytokine responses, without diminishing their avid phagocytic response to bacterial challenge^31^. Modulation of macrophage TNF-α secretion by fibroblasts is enhanced by contact, and can be partially inhibited by blocking both IL6 and IL10. It is unlikely that TGFβ alone plays a major role in the inflammomodulatory activity, since TGFβ is produced by the HGF-1 cell line that is significantly less inflammomodulatory. PDLF also reduced human monocyte migration towards an SDF-1α gradient in a transwell chamber in a contact-independent manner. In contrast, fibroblasts that undergo sub-lethal stress have been shown to attract monocytes in order to repair DNA damage^32^. Taken together, the findings support a role for periodontal ligament fibroblasts in regulation of the inflammatory infiltrate and the host anti-bacterial response. Since host inflammation promotes a dysbiotic oral microbiota characterized by a shift to anaerobic gram negative periodontal pathogens^33^, downregulation of inflammation may paradoxically prevent dysbiosis^34^.

Since inflammation is more severe, and less responsive to treatment in peri-implantitis lesions (where the PDLF are absent) compared to periodontitis lesions (where the PDLF are present), we originally hypothesized that PDLF would be more inflammomodulatory than fibroblasts from the ginigiva which fail to control inflammation in the setting of peri-implantitis. Furthermore, other groups have implicated fibroblasts in tissue damage in inflammatory diseases^35^, raising the possibility that fibroblasts from the gingiva may have opposing effects to fibroblasts from the periodontal ligament. Comparison of the inflammomodulatory activity of PDLF to a human GF cell-line supported our hypothesis. However, comparison of PDLF to primary GF from several human donors (donor-matched and non-donor-matched comparisons) showed that the inflammomodulatory activity of primary GF was at least as potent as that of PDLF. The diminished inflammomodulatory activity of the HGF-1 cell line is likely multifactorial, and may be related to its clonal origin or its passage number. Furthermore, fibroblasts from inflamed peri-implantitis tissue (PIF) were the most inflammomodulatory of all oral fibroblasts tested. Since the phenotype of primary fibroblasts may remain consistent^36^ or may evolve^37^ over increasing passages and age, we examined the regulatory effect of PIF at both early and late passage numbers. Both conditions led to a similar degree of inflammomodulation (Supplementary Fig. S5), suggesting that PIF maintained “positional memory”^38^ and that their higher inflammomodulation capability may be attributed to the inflammatory nature of the original tissue. In addition, priming of oral fibroblasts with inflammatory cytokines (TNF-α or IL1-β) drove them to be more inflammomodulatory. Taken together, these results suggest that fibroblasts from non-inflamed tissue (such as the periodontal ligament) participate in tissue homeostasis by downregulating the infiltration of immune cells and the inflammatory response of those cells that infiltrate. When oral fibroblasts are exposed to inflammatory products, the inflammomodulatory phenotype is enhanced, presumably as a feedback mechanism to control inflammation. In the setting of peri-implantitis, PIF regulatory activity may either be insufficient to control the inflammatory infiltrate, or may fail due to the haphazard organization of the fibroblasts within the tissue in comparison to the ordered arrangement of the periodontal ligament. In either case, it is likely that distinct populations of fibroblasts emerge during chronic inflammation with varying effects on inflammation. The outcome for immune modulation may be influenced by the anatomical site, the disease status and even the origin of different fibroblasts sub-popoulations within the same anatomical site^36, 39, 40^.

Our results suggest that oral fibroblasts actively shape the inflammatory microenvironment during periodontal inflammation. A better understanding of the environmental stimuli that control their behavior is important in the understanding of the failed resolution of periodontal and peri-implant inflammation, both of which progress to tissue destruction and bone loss.

## Materials and Methods

### Materials

The human THP-1 ATCC TIB-202^TM^ (monocyte-macrophage) and HGF-1 ATCC CRL-2014^TM^ (gingival fibroblast) cell lines were obtained from the ATCC (VA, USA). Phorbol 12 Myristate 13-Acetate (PMA) was used to differentiate the THP-1 monocytes into macrophages. Co-culture assays of human fibroblasts and macrophages were performed using Nunc cell culture plates or Transwell® plates with 0.4 µm Pore Polycarbonate Membrane Insert (Corning, N.Y., USA). In some of the co-culture assays, fibroblasts were primed with recombinant human IL1-β or TNF-α for 3 days prior to co-culture with macrophages. Neutralizing antibodies to IL-6 (clone 1936) and IL-10 (clone 25209), and isotype control (clone 20116) were from R&D Systems (MN, USA).

Human TNF-α, IL1-β and IL-6 ELISA MAX^TM^ kits were from BioLegend (CA, USA). Human IL-10 and TGF-β Ready-SET-Go!® kits were from affymetrix eBioscience (CA, USA). For phagocytosis assays, live bacteria were labeled with Fluorescein Isothiocyanate (FITC). Extra cellular bacteria were quenched with 0.2% Trypan-Blue. 5-(and 6)-Carboxyfluorescein diacetate succinimidyl ester (CFSE) was used to label human monocytes during migration assays. Monocyte migration was induced using a gradient of Stromal-Cell Derived Factor-1 (SDF-1α) in a 6.5 mm Transwell® with 5.0 µm Pore Polycarbonate Membrane Insert (Corning). Primary PDLF or GF from human donors were stained with FITC labeled anti-human TGFbRII (clone 25508) antibody or isotype control (clone11711) (R&D Systems).

### Tissue explants

Experiments involving human tissues were conducted according to the approval of the Helsinki committee of the Hadassah– Hebrew University Medical Center, and all donors provided informed consent. Primary PDLF, GF and peri-implantitis fibroblasts (PIF) were obtained from tissue explants of extracted teeth and gingival fragments of 1 mm^3^ from adult volunteers with no signs of periodontal disease (in the case of PDLF and GF), or with peri-implantitis (in the case of PIF), according to the method of Sommerman^41^. Briefly, the explanted tissues were washed 3 times in DMEM containing penicillin (1000 units/ml), streptomycin (1 mg/ml), and amphotericin B (2.5 µg/ml). PDLF were scraped of the middle portion of the root while the gingivae from healthy teeth, or from inflamed peri-implant tissue, were minced into small pieces (1–2 mm^3^). PDLF, GF and PIF explants were allowed to attach separately to culture flasks previously coated with fetal calf serum. When cells began migrating from the explants, culture media was replaced every 3–4 days. Confluent cells were passaged by trypsinization. Fibroblasts were used between the 5^th^ and 10^th^ passage.

### Cell culture

All cells were grown in media consisting of DMEM supplemented with FCS (10%), 4 mM L-glutamine, 1 mM sodium pyruvate, penicillin (100 units/ml) and streptomycin (0.1 mg/ml).

### Bacteria

*Porphyromonas gingivalis* (*P*. *gingivalis*) 381 were cultured in Wilkins media (Oxoid, Basingstoke, UK) under anaerobic conditions (85% N2, 5% H2 and 10% CO2) at 37 °C.

### Co-culture assays

PMA differentiated THP-1 macrophages were seeded in 96 well plates (5 × 10^4^/well) either alone, or with fibroblasts (at 2.5 × 10^4^–1 × 10^5^), or with fibroblast pre-conditioned (pc)-medium. Cells were then stimulated with increasing multiplicity of infection (MOI) of heat-killed (HK) *P*. *gingivalis* 381 for 5 h (37°). Supernatants were collected and analyzed for cytokine secretion by ELISA according to manufacturer’s instructions. To analyze mRNA levels, cells were lysed with TRI-Reagent (Sigma Aldrich, Rehovot, Israel) according to the manufacturer’s instructions. In some of the experiments, fibroblasts were primed with recombinant human IL1-β or TNF-α (10 ng/ml) for 3 days prior to co-culture with human macrophages. In other co-culture assays, we tested the effect of IL-6 and IL-10 neutralization using human IL-6 antibody, human IL-10 antibody or the corresponding isotype control at 10 µg/ml.

### Analysis of mRNA levels by real time quantitative polymerase chain reaction

Total RNA was isolated from each well of the co-culture assays separately, using TRI-Reagent (Sigma Aldrich, Rehovot, Israel) according to the manufacturer’s instructions. The RNA was quantified by spectrophotometry and its integrity was assessed by agarose gel electrophoresis. Reverse transcription of messenger RNA (mRNA) to single strand complementary DNA (cDNA) was carried out with 1 μg of the total RNA sample, using Oligo (dT)18 primer (Thermo Maxima H minus First strand cDNA synthesis kit; Thermo Scientific, MA, USA). Real-time quantitative PCR (qRT-PCR) analysis of the resulting cDNA was performed in a CFX96 Real Time System (Applied Biosystems, CA, USA). The reaction was carried out in a 10 μl reaction volume that contained 5 μl of KAPA2G Fast SYBR Green ReadyMix (Kapa Biosystems, MA, USA), 200 nM each of the forward and reverse primer, and 3 μl of diluted cDNA. The appropriate dilution of cDNA was calibrated for each primer couple. The thermal profile for SYBR Green real-time PCR was 50 °C for 1 minute, then 95 °C for 10 minutes, followed by 40 cycles of 95 °C for 15 seconds, 60 °C for 30 seconds and 72 °C for 30 seconds. The PCR was terminated with 72 °C for 3 minutes followed by a production of a melt curve by increasing from 65 °C to 95 °C at 0.5 °C increment every 5 seconds. Human beta actin (*HBA*) primers were used as an internal control for the analysis of *TNFA*, *IL1B*, *TIA1* and *TTP*. The primers that were utilized are described in Table 1. The results are shown as relative quantity of the mRNA at MOIs 10 and 100 compared to MOI 0 for each culture type separately (Fig. 2a–d).

**Table 1:** Primer sequences used for analysis of the levels of inflammatory mediators.

	Forward (5′ to 3′)	Reverse (5′ to 3′)
*HBA*	CACGGCATCGTCACCAACT	TGATCTGGGTCATCTTCTCGC
*TNFA*	CCCAGGGACCTCTCTCTAATCA	AGCTGCCCCTCAGCTTGAG
*IL1B*	GCTGGAGGACTTTAAGGGTTAC	GATGTCTGGGTCTTGGTTCTC
*TIA1* ^42^	GCCCAAGACTCTATACGTCGGTAACC	GGTGCAAAAGCAGCTTTTATATCTTC
*TTP* ^43^	CATGGCCAACCGTTACACC	AGCGACAGGAGGCTCTCGTAC

The following primer sequences were used to assess the mRNA levels of inflammatory mediators in the co-culture assays, via qRT-PCR.

### Phagocytosis assay

THP-1 macrophages were seeded in 96 well plates (5 × 10^4^/well) either alone or with PDLF (at 2.5 × 10^4^). Cells were exposed to FITC-labeled live *P*. *gingivalis* 381 at MOI 10 for up to 105 minutes. Following incubation, cells were extensively washed in PBS, and treated with 0.2% trypan-blue in PBS to quench any remaining extracellular bacteria. Trypan-blue was removed by an additional wash, and phagocytosis was measured using a fluorescent plate reader.

### Migration assay

CFSE (5 mM)-labeled THP-1 monocytes were allowed to migrate in a 5 µM pore size transwell chamber system towards a gradient of SDF-1α (100 ng/ml). When indicated, PDLF (10^5^) were pre-incubated in the bottom part of the chamber. After 3 h of migration, cells were collected from the top and bottom parts of the chamber, and CFSE labeled cells were counted using a flow cytometer. The fold induction in migration of monocytes toward the SDF-1α gradient was measured, and compared to spontaneous monocyte migration in the absence of SDF-1α.

### Flow cytometry

10^5^ Primary PDLF or GF from human donors were stained with FITC-labeled anti-human TGFbRII antibody or isotype control for 30 minutes on ice. Cells were washed in PBS 2% FCS. The percent of FITC positive cells was measured using a flow cytometer.

### Alkaline phosphatase (ALP) activity assay

ALP activity assay was performed as previously described^44^ in a colorimetric assay using fibroblasts seeded in 96 well plates (5 × 10^3^–2 × 10^4^ cells/well) and cultured for 4 days.

### Statistical analysis

Results are based on the use of at least 5 donors of primary PDLFs and 5 donors of primary GFs. Mean and SEM are used for descriptive statistics. Results of co-culture experiments are based on at least 3 biological repeats. In order to determine the effect of co-culture and the MOI used in the co-culture experiments, we used 2-way analysis of variance (ANOVA). Furthermore, multiple pairing analyses were done with Mann-Whitney test and Bonferroni post-test for significance. All statistical analyses were two tailed and a P value of 0.05 or less was considered statistically significant.

## Electronic supplementary material

Supplementary data
