# Supplementary material for: Oral fibroblasts modulate the macrophage response to bacterial challenge
Source: Sci Rep. 2017 Sep 14;7:11516. doi: 10.1038/s41598-017-11771-3 (PMC5599598; doi:10.1038/s41598-017-11771-3)
Supplement: Supplementary file 1 — Supplementary data [file 41598_2017_11771_MOESM1_ESM.doc]

**Title**:

Oral fibroblasts modulate the macrophage response to bacterial challenge

**Authors**:

Rinat Tzach-Nahman^a,b^, Rizan Nashef^c^, Omer Fleissig^a,d^, Aharon Palmon^a^, Lior Shapira^b^, Asaf Wilensky^b,*^ and Gabriel Nussbaum^a,*^

**Institute**:

^a^ The Institute of Dental Sciences, ^b^ The Department of Periodontology , ^c^ The Department of Oral and Maxillofacial Surgery, ^d^ The Department of Orthodontics and the, the Hebrew University-Hadassah Faculty of Dental Medicine, Jerusalem, Israel. *These authors contributed equally.

**Short title:** Oral fibroblasts modulate inflammation

**Corresponding author:**

Gabriel Nussbaum, Institute of Dental Sciences, Faculty of Dental Medicine, Hadassah

Medical Center, Hebrew University, Jerusalem, Israel.

Telephone number: +972-2-6758581; Fax number: +972-2-6758561

E-mail address: [gabrieln@ekmd.huji.ac.il](mailto:gabrieln@ekmd.huji.ac.il)

**Keywords**: Fibroblasts; Porphyromonas gingivalis, Inflammation, Macrophages, Human

**
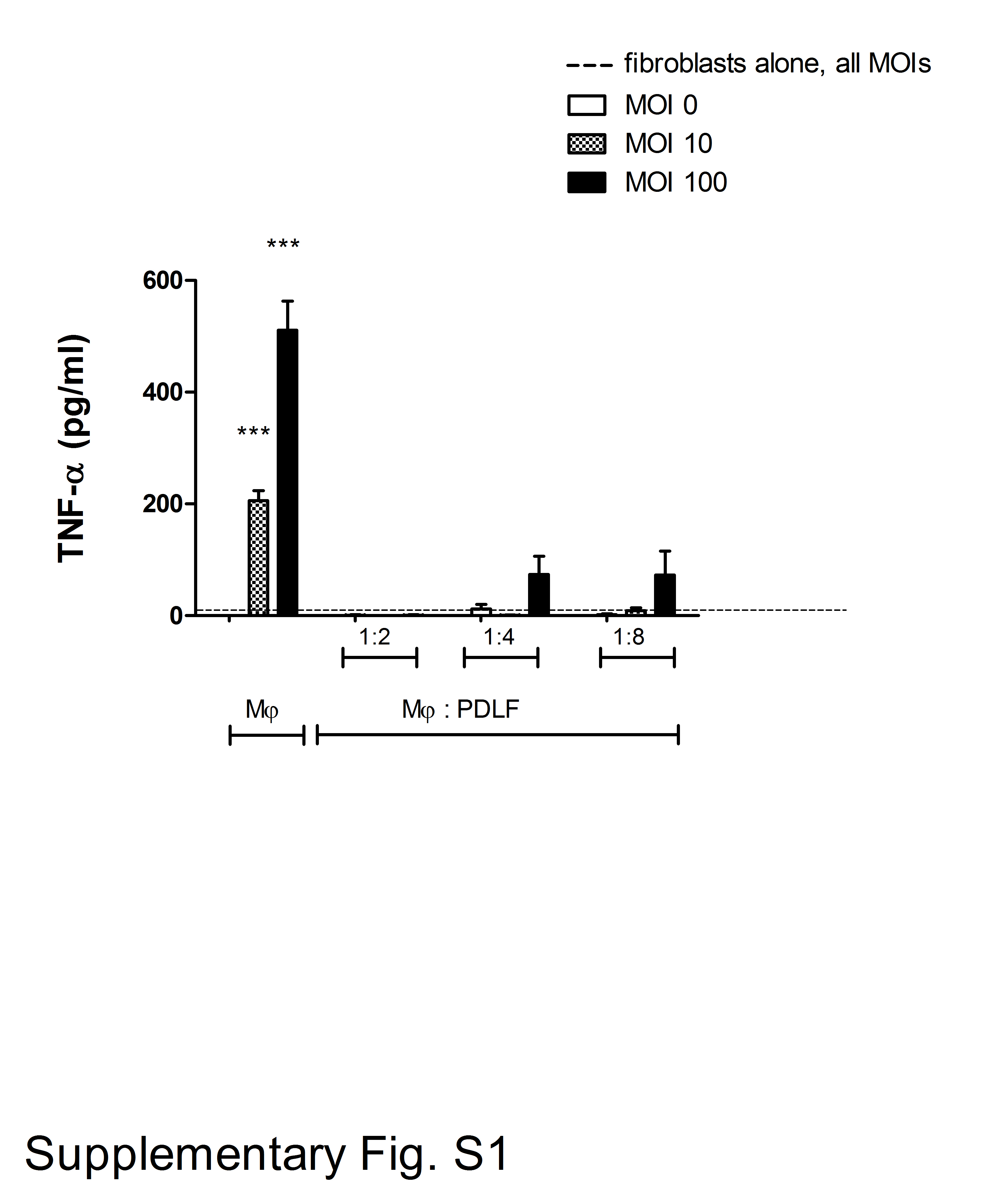
**

**Supplementary Fig. S1**

**PDLF down-regulate macrophage cytokine production in response to *P. gingivalis*.**

Human macrophages (Mϕ) were mono or co-cultured with PDLF at different cell to cell ratios. Cells were stimulated with increasing MOIs of *P. gingivalis* and TNF-α production was measured in the supernatant 5 hours after bacterial stimulation. *** P value <0.01 compared to the cytokine production by Mϕ in mono-culture stimulated with the same MOI.

**
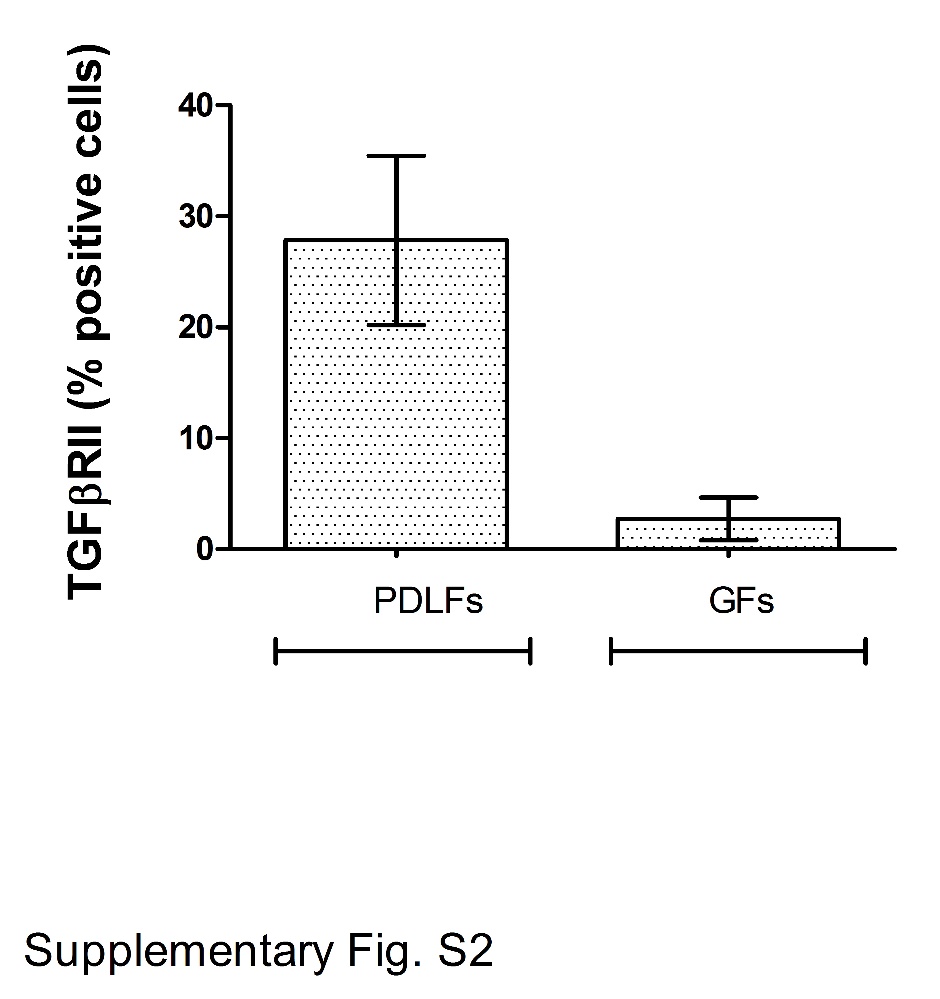
**

**Supplementary Fig. S2**

**Primary PDLF express higher amounts of TGFβRII than donor and non-donor matched primary GF**

Pairs of primary human PDLF and GF were stained for TGFβRII in order to validate extraction of two different populations of fibroblasts. The percentage of TGFβRII positive cells was evaluated by flow cytometry in comparison to cells stained with a matching isotype control antibody. Data represent the means + SEM of 3 different donors of each cell type.

**
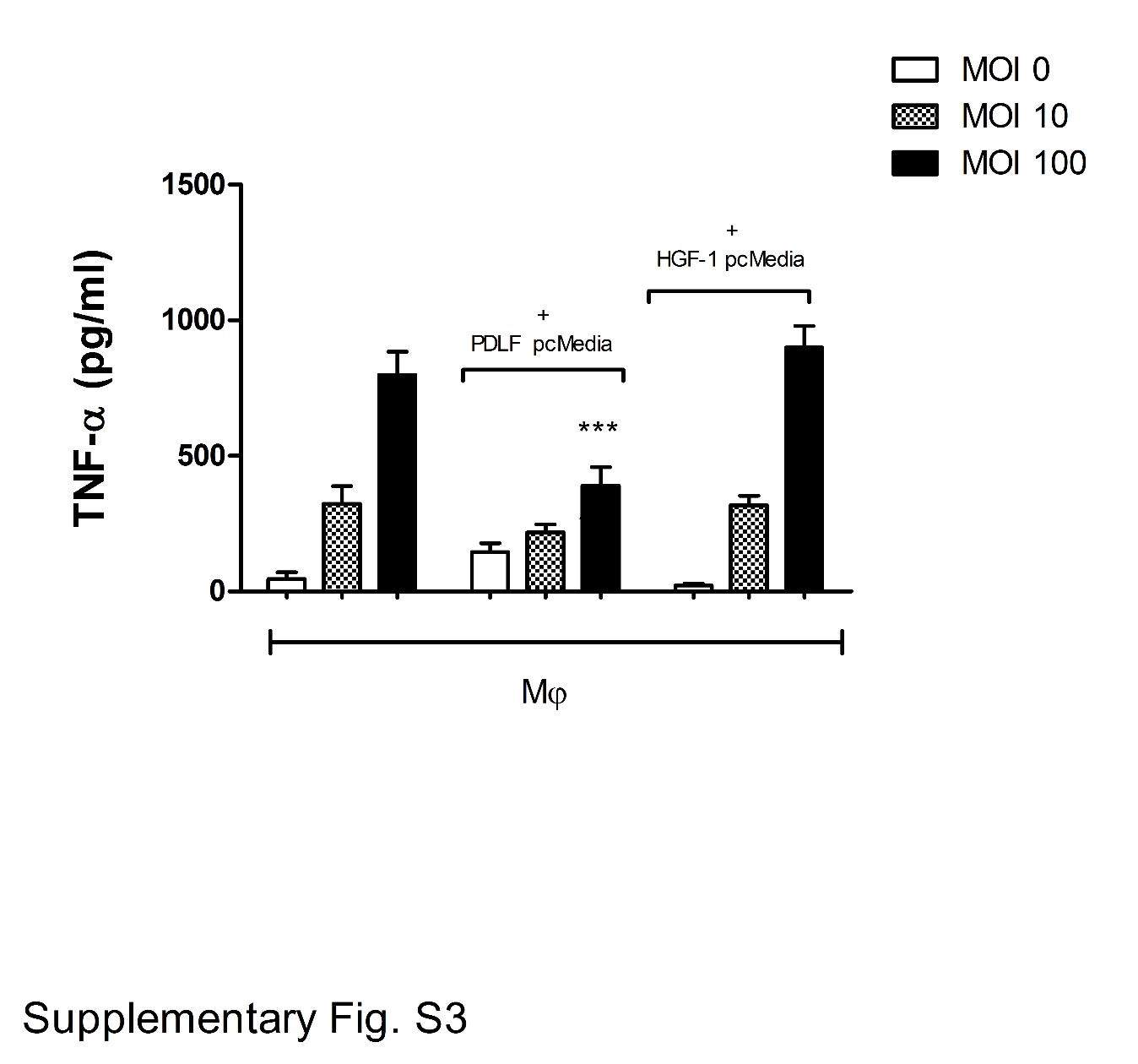
**

**Supplementary Fig. S3**

**pcMedia of PDLF but not HGF-1 down-regulate macrophage cytokine production in response to *P. gingivalis*.**

Human Mϕ were mono or co-cultured with pcMedia derived from naive PDLF or HGF-1. Cells were stimulated with increasing MOIs of *P. gingivalis* and TNF-α production was measured in the supernatant 5 hours after bacterial stimulation. *** P value <0.01 compared to the cytokine production by Mϕ in mono-culture stimulated with the same MOI.

**
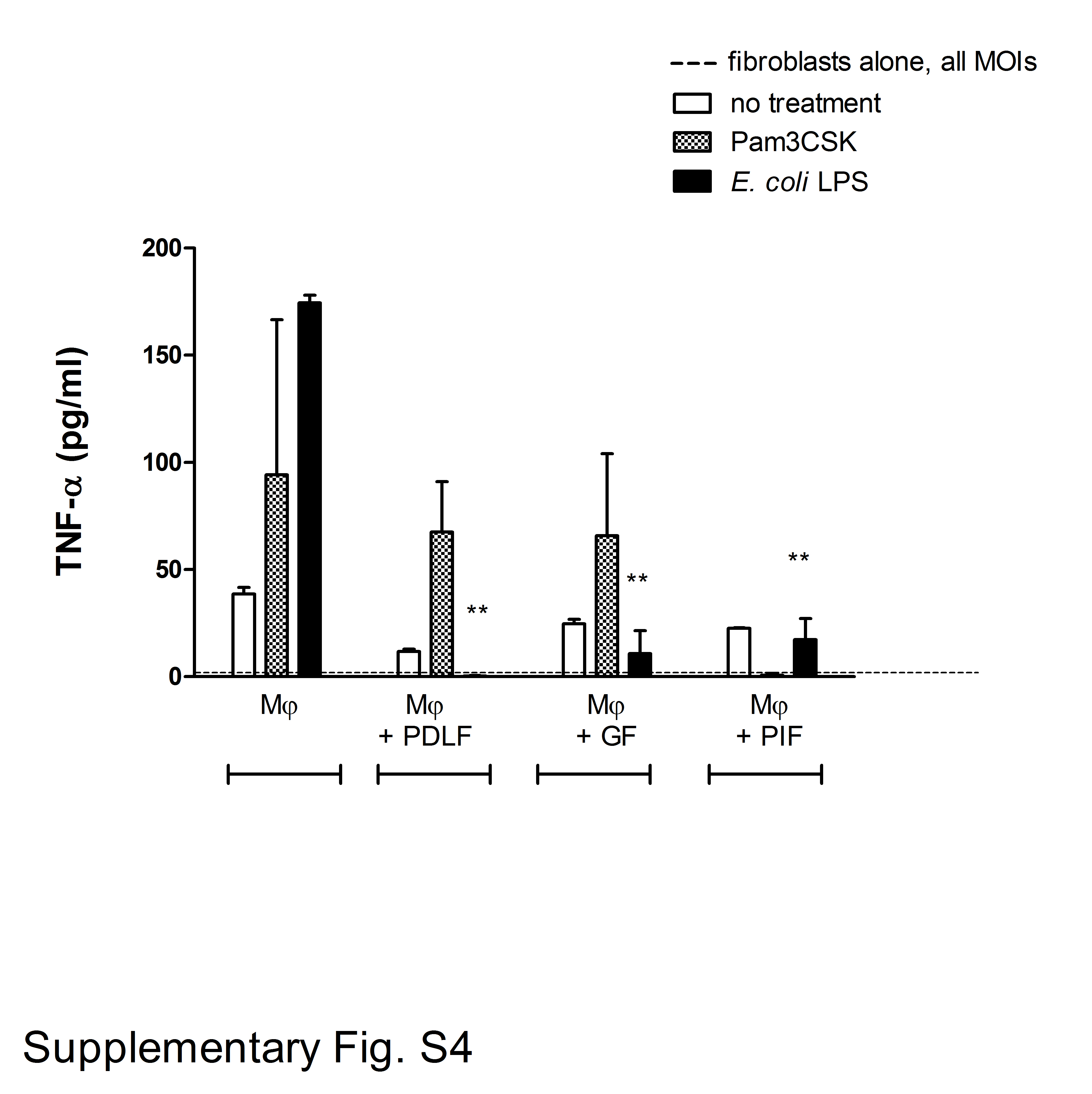
**

**Supplementary Fig. S4**

**Fibroblasts modulate the innate inflammatory response of Mϕ non specifically**

Human Mϕ were mono or co-cultured with PDLF, GF or PIF. Cells were stimulated with different TLR ligands to examine whether fibroblasts modulate macrophage response to *P. gingivalis* specifically or not. ** P value <0.01 compared to the cytokine production by Mϕ in mono-culture stimulated with the same treatment.


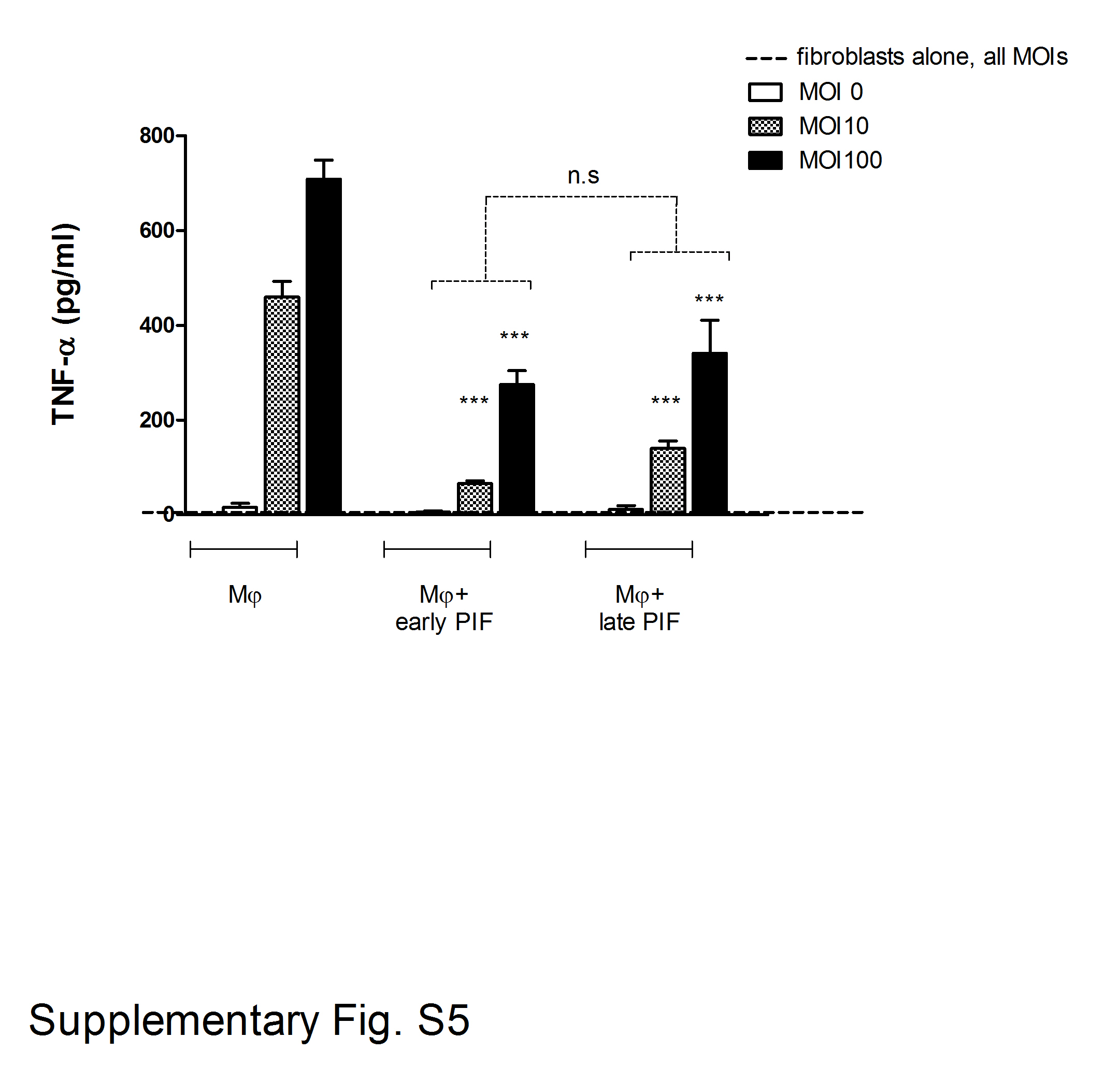


**Supplementary Fig. S5**

**PIF maintain their inflammomodulation ability at both early and late passages**

Human Mϕ were mono or co-cultured with early and late passage PIF. Cells were stimulated with increasing MOIs of *P. gingivalis* and TNF-α production was measured in the supernatant 5 hours after bacterial stimulation. Protein levels were compared between culture types at the same MOI. *** P <0.001, n.s= not significant.

n.s = no significant differences in cytokine production by co-cultures with early or late PIF, stimulated with the same MOI.
